# Development of three new multidimensional measures to assess household food insecurity resilience in the United States

**DOI:** 10.3389/fpubh.2022.1048501

**Published:** 2022-12-14

**Authors:** Eric E. Calloway, Leah R. Carpenter, Tony Gargano, Julia L. Sharp, Amy L. Yaroch

**Affiliations:** ^1^The Gretchen Swanson Center for Nutrition, Omaha, NE, United States; ^2^Graybill Statistical Laboratory, Colorado State University, Fort Collins, CO, United States

**Keywords:** household resilience, social determinants, food security, financial wellbeing, absorptive capacity, adaptive capacity, Transformative Capacity

## Abstract

**Introduction:**

This study aimed to develop and test novel self-administered measures (Absorptive capacity, Adaptive capacity, and Transformative capacity) of three aspects of a household's resilience to financial shocks (e.g., job loss) that can increase food insecurity risk.

**Methods:**

Measures were piloted in a convenience sample of households at risk for food insecurity in the United States. The survey included the new measures, validation variables (financial shock, household food security, general health, personal resilience to challenges, and financial wellbeing), and demographic questions. Exploratory factor analysis was used to assess dimensionality, internal consistency was assessed [Cronbach's alpha (CA)], and construct validity was assessed (Spearman's correlation). Also, brief screener versions of the full measures were created.

**Results:**

Participants in the analytic samples (*n* = 220-394) averaged 44 years old, 67% experienced food insecurity, 47% had a high school diploma or less, 72% were women, and the sample was racially/ethnically diverse. Scores for Absorptive capacity [one factor; CA = 0.70; Mean = 1.32 (SD = 0.54)], Adaptive capacity [three factors; CAs 0.83-0.90; Mean = 2.63 (*SD* = 0.85)], and Transformative capacity [three factors; CAs 0.87-0.95; Mean = 2.70 (*SD* = 1.10)] were negatively associated with financial shocks (−0.221 to −0.307) and positively associated with food insecurity (0.310-0.550) general health (0.255-0.320), personal resilience (0.231-0.384), and financial wellbeing (0.401-0.474).

**Discussion:**

These findings are encouraging and support reliability and validity of these new measures within this sample. Following further testing, such as Confirmatory Factor Analysis in future samples, these measures may prove useful for needs assessments, program evaluation, intake screening, and research/surveillance. Widespread adoption in the future may promote a more comprehensive understanding of the food insecurity experience and facilitate development of tailored interventions on upstream causes of food insecurity.

## Introduction

Household food insecurity (defined as “access by all people at all times to enough food for an active, healthy life”) rates in the United States (U.S.) have hovered around 10–15% (1) since the U.S. Department of Agriculture (USDA) started tracking it in a uniformed way utilizing the Household Food Security Survey Module (HFSSM) in 2000 (2). Food insecurity is associated with poor dietary and physical and mental health outcomes (3–11). Food insecurity also disproportionately affects households with lower incomes, those that are headed by single individuals, people from racial/ethnic minority groups, parents with young children, and/or households located outside metropolitan areas (e.g., rural) (2). Further, during the COVID-19 pandemic, many households faced financial challenges and food insecurity that they may not have experienced before. In order to develop programming and policy interventions to address food insecurity, we need to better understand antecedents of this condition.

One cause of food insecurity for many households is experiencing household-level financial shocks (12). These financial shocks include unexpected losses in income (e.g., job loss, reduced hours, decreased business profitability) and unexpected large expenses (e.g., medical emergency, automobile accident, necessary home repair). Financial shocks cost households between $500 and 2,000 on average (12–15) and impact between 25 and 60% of households each year (15, 16), Following a financial shock, it can take some households ~6 months to regain financial stability, if at all (15). Financial shocks affect low- and middle-income households disproportionately (17) due to typically limited liquid assets and tight budgets (3, 16, 18). Lower income households who experience financial shocks may have to forgo medical care, skip bills, and reduce food intake (12, 19–21). For households below 200% of the federal poverty level, experiencing a $500 financial shock is associated with a 20% increased odds of food insecurity (12). Clearly, understanding financial shocks and what makes a household susceptible to them may be crucial for developing tailored food insecurity interventions.

The socio-ecological resilience literature offers a way to frame a household's vulnerability to financial shocks. Socio-ecological resilience literature has typically focused on large adaptive systems (e.g., cities) and their response to a disturbance (e.g., a hurricane) (22). However, relatively recently, Alinovi et al. (23) posited household itself could be viewed as a complex adaptive system, with financial shocks as the disturbance, especially in the context of food insecurity. Household resilience in the context of food insecurity has been defined several ways. For instance, Alinovi et al. (23) writes, “…household resilience to food insecurity… is defined as a household's ability to maintain a certain level of well-being (food security) in the face of risks, depending on that household's available options to make a living and its ability to handle risks”.

In subsequent refinements of the Alinovi et al. (23) model, researchers (24–27) have outlined the three primary constructs or “capacities” that define a household's resilience to financial shocks that might increase food insecurity risk—Absorptive, Adaptive, and Transformative Capacities. Absorptive Capacity is the “ability of the [household] to minimize its exposure to shocks, but also having mechanisms to recover quickly when shocks actualize” (24). Adaptive Capacity is the “the ability of the [household] to make informed choices about alternative livelihood strategies based on changing conditions” (24). Transformative Capacity is the “conditions that are necessary for changing the basic configuration of the [household] to create long-term resilience” (24).

**Figure 1:**
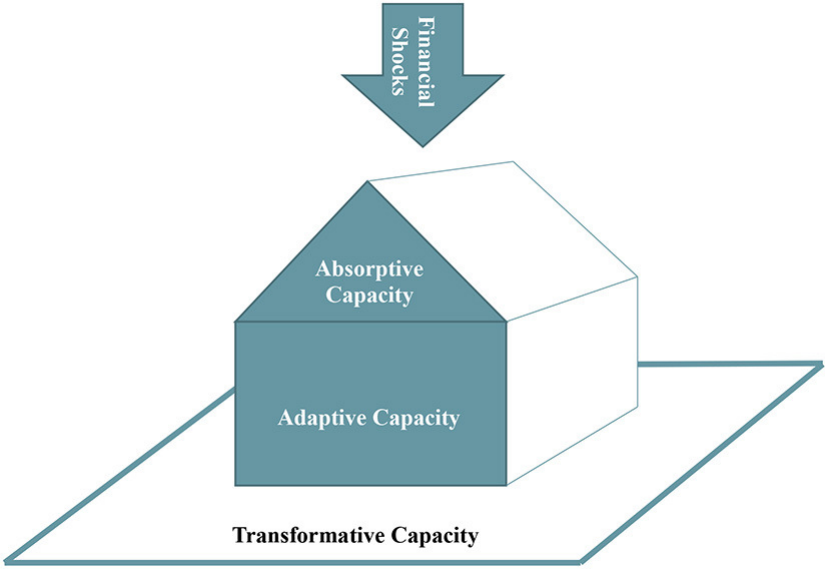
The relationships between financial shocks absorptive, adaptive, and transformative capacity. At the top is a downward arrow representing financial shocks that a household may face (e.g., unexpected large medical bills, a car wreck damaging the family car, a main earner for the household losing their job) that may put the household in financial jeopardy and risk for food insecurity. For absorptive capacity, on-hand resources are used initially to buffer the shock (similar to how a roof protects a house). If the shock cannot be buffered, then the household must use adaptive capacity, such as skills, efficacy, knowledge, to react and adapt to the shock (represented in this model as the core of the house where the people live). If a household cannot react and adapt, they may be at risk for food insecurity. Transformative capacity refers to the conditional factors related to the community the household is situated in that limit or support a household's ability to transform their resilience in the long-term (represented in the model as the area surrounding the house).

These capacities are themselves multidimensional and assess three different perspectives on the same latent phenomenon—household resilience (25). They represent increasing time components (short-, intermediate-, and long-term, respectively) and increasing “robustness” toward shocks (25). When faced with a financial shock, a household uses on-hand resources to “Absorb” the impacts of the shock in the short term. If a household's Absorptive Capacity to buffer a shock is exceeded, the household must react and adjust livelihood strategies to “Adapt” to the shock in the intermediate term (25, 28). Then, if eventually adaptation required to handle the shock becomes too great, the household will need to “Transform” in the long-term (25). For a household to be able to transform, changes in macro factors (e.g., policies, systems, and environments) may be needed. For example, a member of a household may need to alter career paths for better wages, but if training or job opportunities are not available in their area, their Transformative Capacity may be limited. Different types of resilience variables are relevant to each capacity. On-hand resources are more relevant to Absorptive Capacity, knowledge and skills to Adaptive Capacity, and community-level conditions to Transformative Capacity. Also, the types of interventions likely required to influence each capacity varies. See Figure 1, below that helps illustrate these capacities and how they related to each other.

The extant literature investigating these three household resilience capacities and their relationship with food insecurity has focused mainly on lower-income countries in Africa, Asia, and South America (24). For example, Smith and Frankenburger (29) found that flood-impacted households in northern Bangladesh who had higher pre-flood household resilience scores were less likely to experience post-flood food insecurity (29). Variables they found to be especially important to household resilience were mapped to the three capacities model and included factors such as assets/savings, bonding social capital, and disaster preparedness (Absorptive Capacity); confidence to adapt, human capital, exposure to information (Adaptive Capacity); and linking/bridging social capital, access to markets, governance (Transformative Capacity). Additional resilience variables observed in lower-income countries included household income diversity, informal safety nets (e.g., help from friends or family), long-term outlook/aspirations, access to public services and infrastructure, community and institutional environment, environmental/ecological factors, and issues of basic human rights and empowerment (23, 24, 27, 29).

Greater household resilience has been shown to be associated with increased food security and decreased malnutrition (29–31). However, factors relevant to household resilience can be highly context dependent (23, 26, 32). The specific resilience variables observed in one country may or may not be relevant or translate to household resilience in another. The household resilience food security literature has predominately focused on countries outside the U.S., such as those with lower median incomes and agriculturally based economies where households' livelihoods are more susceptible to natural hazards. Further, there has not been a standard accepted self-administered measure to assess household resilience or evaluate resilience building programs (27). While some progress toward this end has been made in lower income countries, there has been limited, if any, work done in higher income countries who also face food insecurity. Such a tool could be beneficial to organizations who work in the U.S. to implement and evaluate food security-focused programmatic and policy interventions that aim to build household resilience to financial shocks.

The purpose of this study was to conduct the preliminary development of U.S.-relevant self-administered measures to assess household Absorptive Capacity, Adaptive Capacity, and Transformative Capacity. Further, the tools were intentionally developed to be easy to administer and score, and to provide practical data and information (in addition to a quantitative score) to inform program implementers.

## Methods

### Study overview

From January 2020 to December 2021, the study authors sought to identify food-insecurity-related measurement gaps and develop measures to address those gaps. There were three measurement gaps addressed in the overall study, this paper reports on development and validation of measures to assess one of those gaps—household resilience. The work was completed in two main phases. First, a formative phase focused on identifying the measurement gap and developing item pools to address the gap. Second, a testing phase included administering a pilot survey and performing psychometric analyses. Exploratory factor analysis was performed and construct validity was assessed. Also, brief “screener” versions of the measures were identified. Analyses were conducted using SAS version 9.4. The study application was reviewed by the University of Nebraska Medical Center Institutional Review Board and the study authors were authorized to begin research. Interviewees provided oral informed consent and survey respondents provided written informed consent. All prevailing ethical standards in protecting human subjects were followed.

### Formative phase overview

We will describe the formative work briefly and refer the readers to Calloway et al. (33) for a more complete account. The purpose of the initial formative steps (January 2020–January 2021) was to create testing-ready pools of items that would be used for the testing phase. The inputs for this phase included an Expert Advisory Group (EAG), literature scans, and formative and cognitive interviews. The EAG [university researchers (*n* = 7), food insecurity non-profits leaders (*n* = 6), and federal government staff (*n* = 1)] helped prioritize measurement gaps and items, refine operational definitions, and provide advice on testing plans. Next, two iterative literature scans were conducted to identify and classify existing survey items that could be used and modified. Also, new items were created as needed. All candidate items were presented to the EAG. Following the literature scans, semi-structured 60-min formative interviews (*n* = 47; 42 in English, five in Spanish) were conducted with adults experiencing food insecurity or at risk for food insecurity across five states (AR, CA, MD, NE, and TN) to understand their experiences and ensure the selected items were relevant. Items were then arranged into draft versions of the new measures—ready for cognitive interviewing. A total of ten cognitive interviews were conducted from December 2020 to January 2021 with adults (one man and nine women) experiencing food insecurity or at risk for food insecurity in California (*n* = 8) and Nebraska (*n* = 2). Interviews last about 60 min and employed a “think aloud” technique in which participants explained their thought process while answering questions in the draft survey (34). Revisions were made to make wording easily interpretable, reduce cognitive burden, and to prioritize or delete items.

### Testing phase

#### Piloting the survey

Survey items resulting from the formative phase were tested in samples recruited with the help of partner organizations (different partners than the formative phase) across five states (CA, FL, MD, NC, and WA). A survey was created for pilot testing which included items for the new measures, scales and items needed for validation, and demographic questions. Partner organizations (*n* = 7) that worked with households at risk for or experiencing food insecurity (e.g., food pantries, shelters, resource centers, etc.) across the aforementioned five states recruited survey participants from April to June 2021. Inclusion criteria were that the respondent was at least 18 years old, understood English, could answer questions about themselves and the household, and was from a household experiencing food insecurity or at risk for food insecurity. The partner organizations were asked to recruit a total of ~200 respondents per state, and sample diversity was monitored for race/ethnicity, age, gender, household composition (e.g., with/without children, single adults, cohabitating adults, etc.), rurality, and gradients of income across lower income levels with an aim to ensure sample diversity was similar to the populations the sites served. Recruitment and data collection procedures were tailored to each site. Recruitment occurred *via* email, texting, and/or flier. In order to reach more of the populations served at each data collections site, sites advised the research team to offer both paper and web-based survey options and sites utilized these survey modes based on the needs of their participants (test bias by survey mode was investigated in the psychometric analyses). Pilot surveys contained ~75–85 items each including items for the new measures, validation variables (i.e., financial shock, household food security, general health, personal resilience to challenges, and financial wellbeing), and demographic questions. One survey was completed per household and respondents received $25 gift cards for completing the survey.

#### Survey variables

In addition to the items for the new measures, the following variables were included in the pilot survey and used in the analyses to assess convergent and discriminant validity.

##### Financial shock

To assess experiencing a financial shock, respondents answered the following question, “In the last 12 months, has your household had any large expenses (e.g., unexpected medical bills, car repairs) or large declines in income that you did not expect?” Response options were “Yes” or “No” and scored 1 or 0, respectively.

##### Household food security

The USDA HFSSM, 6-item 30-day version, was used to assess household food security (35). Households were assigned food security categories based on the number of affirmative (i.e., “Sometimes true” or “Often true”) responses using the standard approach (35) to produce a four-level ordinal variable for the analyses—very low food security, low food security, marginal food security, and high food security. The 6-item version was used to reduce respondent burden. The 30-day version was used because it matched more closely to the candidate household resilience items that assess current perceived Absorptive, Adaptive, and Transformative Capacity.

##### General health

Self-reported general health was assessed using an item from the Centers for Disease Control and Prevention's (CDC) Behavioral Risk Factor Surveillance System (BRFSS) survey (36). Respondents rated their general health from “Poor” (Scored as a 1) to “Excellent” (Scored as a 5). This was treated as a five-point ordinal score for the analyses.

##### Personal resilience

The two-item version of the Conner-Davidson Resilience Scale (CD-RISC) (37) was administered with 5-point ordinal response options ranging from “Not true at all” (scored 0) to “True nearly all of the time” (scored 4). The sum of the two items was the scale score. This scale was developed to assess individuals' responses to general challenges, especially as they relate to stress response, engaging others for support, self-efficacy, and other related concepts.

##### Financial wellbeing

The five-item version of the Consumer Financial Protection Bureau's Financial Wellbeing Scale (CFPB FWS) (38) was administered with 5-point ordinal response options (Scored 0–4, with negatively worded items reverse scored, per CFPB guidance). The sum of the items' scores was the scale score. This scale was developed to assess “financial well-being”, which was defined by the developers as, “…a state of being wherein a person can fully meet current and ongoing financial obligations, can feel secure in their financial future, and is able to make choices that allow them to enjoy life”.

##### Sports escapism

An item from a scale (39) assessing sports escapism (e.g., using sports as a past time to distract from usual day-to-day activities) was included to assess discriminant validity. The item chosen was: “Keeping up to date with sports provides an escape from my day-to-day activities” and response options were a seven-point Likert scale from “Strongly disagree” (scored as 1) to “Strongly agree” (Scored as 7). This item was modified to remove the original “Basketball” and replace it with “Keeping up to date with sports…” This item was chosen because it was not conceptually related to diet or moderators of diet (e.g., socioeconomic status) and was from a scale shown not to be associated with gender (and the item score was confirmed in this study not to differ significantly by gender). Responses to this question were treated as a seven-level ordinal variable for analyses.

#### Scoring the newly developed household resilience measures

For a full explanation of the scoring approach, see the Supplementary Tables 1–3 or at the website https://www.centerfornutrition.org/food-insecurity-measures.

##### Absorptive capacity

The measure is a mean score of the items and can range from 0 to 3. The measure assesses household-level income stability, housing stability, and perceived financial wellbeing. Higher scores indicated higher Absorptive Capacity.

##### Adaptive capacity

The measure is a mean score of the items and can range from 0 to 5. The measure assesses household-level perceived financial self-efficacy, perceived financial knowledge and skills, access to information and intangible social support, financial worry/stress, and adaptive barriers (i.e., job barriers and barriers to utilizing governmental assistance programs). Higher scores indicated higher Adaptive Capacity.

##### Transformative Capacity

The measure is a mean score of the items and can range from 0 to 5. The measure assesses community-level perceived access to opportunities, community services/infrastructure, neighborhood cohesion, and household-level future financial outlook (in the next 5 years). Higher scores indicated higher Transformative Capacity.

#### Data cleaning and assessing missing observation percentages

A total of 519 surveys were at least 70% completed. Of these, three duplicate households were removed. “Speeders” (*n* = 10) who completed the survey at a rate that would likely indicate they were being inattentive (i.e., reading faster than 450 words per minute) and “straightliners/skippers” (*n* = 18) who skipped and/or selected only one of the response types for most of the survey items were removed (40). Also, items with excessive missing responses (i.e., a combination of skipped, “don't know,” or “not applicable”) were removed. For items that asked about the household and/or individual, if ≥15% of responses were missing, then it was removed. For items asking about perceptions of the community that the household is situated in, higher missing percentages were expected (≥25% was the cut off for these items). Items AB3 (36.7%), AB4 (20.7%), and TR11-14 (28.1–37.1%) had percentages of missing observations above the threshold and were removed. The abbreviation AB refers to absorptive capacity items, AD refers to adaptive capacity, and TR refers to transformative capacity. Items are numbered to give each a unique identifying code. The unique item codes and full item wording can be seen in the Supplementary material. Participants with complete data for the remaining items were included in the analytic sample, and there was an analytic sample for each of the three new measures.

#### Psychometric assessment

Unweighted least squares exploratory factor analysis with quartimin (oblique quartimax) rotation was performed. Decisions on item inclusion were based on quantitative testing metrics, and qualitative assessment of each measure's ability to provide actionable data that could inform intervention approaches and/or needs assessments. Items that did not load unambiguously (i.e., factor loading <0.4) to one of the factors were removed (41) unless the item provided valuable practical information and loaded more than 0.3, then it may have been retained. A holistic assessment of scree plots, eigenvalues, and conceptual meaning were used to determine the number of factors to extract. Cronbach's alpha was used to assess internal consistency of the measures/scales, with ≥0.70 used as an acceptable standard (42). Lastly, test bias was assessed by examining changes in the magnitude of the relationship between the new measures' scores and a variable they are theoretically associated with (e.g., food security status). Analysis of variance (ANOVA) using general linear models were used to examine potential test bias by assessing moderation of the relationship between the new measures' scores and food security status by race, gender, age, education, and survey mode (i.e., online vs. paper survey). Statistically significant (*p* < 0.05) interaction terms indicated potential test bias (43).

#### Construct validity approach

Spearman's rank correlation was used to assess convergent and discriminant validity by assessing the statistical relationships between the new measures and previously used scales and survey items. It was hypothesized that each of the three new measures should be positively associated with household food security, general health, personal resilience, and financial wellbeing. It was hypothesized that the three new measures should each be negatively associated with having reportedly experienced a “financial shock”. For discriminant validity, it was hypothesized that there should be no association with “sports escapism” for either of the new measures. There were 18 hypotheses being tested (six for each of the three new measures), therefore, the Bonferroni procedure was used to adjust the significance level to 0.0028 (i.e., 0.05/18) to limit the familywise error rate.

#### Determining brief screener versions for the new measures

Brief (e.g., one or two item) versions of the final measures may be necessary for certain applications (e.g., clinical intake screening to inform referrals to assistance programs). To screen for “low” (i.e., below the median measure's score) Absorptive Capacity, Adaptive Capacity, and Transformative Capacity, all single item and all two-item combinations taken from the full version were assessed for sensitivity, specificity, and Cohen's kappa, with higher preference for more sensitive measures. Desirable screening performance was high sensitivity and specificity, and inter-test reliability (Cohen's kappa) of ≥0.6 (44). There are not established standards for what constitutes high sensitivity and specificity, and necessary levels of sensitivity and specificity are context specific (e.g., screening for life-threatening disease vs. intake screening to inform assistance referrals). We chose 85% for sensitivity and 75% for specificity as desirable thresholds for the screeners. These thresholds allow for a precise screening (e.g., a high percentage of all “low” scoring households are correctly identified), but a relatively more moderate threshold for reliably identifying only “low” scoring households (e.g., some households screened as “low” are not “low” for the full measure). These tools will be used for screening households at risk to refer to programs or assistance, rather than medical procedures, and so false positives are not as important as false negatives in this context. Also, the ease of administration was considered with a preference for ordinal and Likert questions, rather than “select-all-that-apply” questions.

## Results

### Formative phase

The EAG identified five key measurement gaps, of which they prioritized the top three—one of which being the assessment of household resilience. Operational definitions were developed after reviewing the scientific literature and in consultation with the EAG. To address the household resilience gap, a total of 171 candidate survey items were reviewed by the EAG, with 79 items ultimately being examined further in the cognitive interviews. This set of 79 items included 21 items for Absorptive Capacity, 22 for Adaptive Capacity, and 36 for Transformative Capacity.

Over two rounds of cognitive interviews, items were modified based on interviewee recommendations. Modifications included wording changes for clarity, streamlining sentences, modifying formatting, and adjusting “select-all-that-apply” lists (e.g., adding, combining, and grouping response options). Interviewees also provided advice about items to cut or add and insight into how they interpreted questions that informed modifications. Following cognitive interviewing, 14 Absorptive Capacity (AB1-AB6, AB7a-c, AB8-12), 18 Adaptive Capacity (AD1-AD12, AD13a-c, AD14a-c), and 21 Transformative Capacity (TR1-TR21) items were included in the pilot survey. See Supplementary Table 4 for final item wording.

### Testing phase

#### Sample characteristics

The analytic sample sizes varied based on complete data: Absorptive Capacity Sample (*n* = 394), Adaptive Capacity Sample (*n* = 325), and Transformative Capacity Sample (*n* = 220). Respondents were ~44 years old on average, most households had children, two-thirds were food insecure, nearly three-fourths were women, and the sample was racially/ethnically diverse. See Table 1 for more sample characteristics broken out for each measure's analytic sample. For “sports escapism”, the discriminant validity variable, the means (SD) were 3.11 (1.87), 3.23 (1.91), and 3.31 (1.97) for the Absorptive Capacity, Adaptive Capacity, and Transformative Capacity samples, respectively. Approximately 70% completed their survey online and the remainder completed a paper survey. Those who were able to participate *via* paper surveys, compared to online surveys, were more likely to be men, be White (non-Hispanic), be above the sample median for age, and not have participated in post high school education (ps < 0.05). However, scores for the new measures did not vary by survey mode.

**Table 1:** Selected sample characteristics for the analytic samples for each of the new measures.

**Sample characteristics**		**Absorptive capacity sample (*n* = 394)**	**Adaptive capacity sample (*n* = 325)**	**Transformative capacity sample (*n* = 220)**
Age (years)	Mean (SD; range)	44.4 (14.7; 18–88)	43.8 (14.2; 18–81)	43.5 (15.1; 18–88)
Proportion of federal poverty level	Mean (SD; range)	0.78 (0.62; 0.06–4.89)	0.79 (0.66; 0.06–3.96)	0.76 (0.64; 0.06–3.96)
CD–RISC^a^	Mean (SD; range)	4.90 (1.85; 0–8)	4.98 (1.83; 0–8)	5.05 (1.92; 0–8)
CFPB FWS^b^	Mean (SD; range)	6.87 (4.19; 0–20)	7.39 (4.23; 0–20)	7.88 (4.39; 0–20)
Experienced financial shock^c^ (%)		63%	62%	63%
Households with children (%)		57%	54%	57%
Women (%)		75%	71%	72%
Food security (%)	High	20%	20%	21%
	Marginal	14%	14%	14%
	Low	29%	29%	28%
	Very Low	37%	37%	37%
Reported general health	Excellent	3%	4%	3%
	Very good	8%	8%	10%
	Good	39%	37%	38%
	Fair	36%	38%	38%
	Poor	14%	13%	11%
Educational attainment (%)	Less than high school	9%	10%	12%
	High school diploma or G.E.D.	34%	32%	30%
	Some college	31%	28%	30%
	Associates degree or greater	26%	30%	28%
Race or ethnicity (%)	White, non-hispanic	48%	50%	52%
	Latino/hispanic	22%	20%	20%
	Black, non-hispanic	17%	19%	16%
	Multi-racial/-ethnic, or another listed	7%	7%	7%
	Asian, non-hispanic	4%	3%	3%
	Tribal/indigenous, non-hispanic	2%	2%	2%
State	California	26%	22%	21%
	Florida	22%	21%	21%
	Maryland	14%	14%	15%
	North Carolina	22%	25%	26%
	Washington	16%	18%	18%

a Conner-Davidson Resilience Scale.

b Consumer Financial Protection Bureau's Financial Wellbeing Scale.

c Self-report of having experienced a household financial shock (e.g., job loss) in the previous 12 months.

#### Exploratory factor analysis, internal consistency, and test bias

Exploratory factor analysis was conducted to examine the factor structure of the candidate sets of items (see Table 2). The Absorptive Capacity measure (Mean score = 1.32; SD = 0.54; max range 0–3) was unidimensional with an Eigenvalue of 1.83 and an acceptable Cronbach's alpha of 0.70. Items in this factor included the overall financial wellbeing and stability of the household. Items AB10-AB12 (which assessed health insurance access, alternative strategies to acquire money, and tangible social support, respectively) did not load onto the factor (i.e., factor loading <0.4) and were removed.

**Table 2:** Exploratory factor analysis showing the factor structures of the three new measures.

**Absorptive capacity (*****n*** = **394)**		**Adaptive capacity (*****n*** = **325)**				**Transformative capacity (*****n*** = **220)**			
	**F1**		**F1**	**F2**	**F3**		**F1**	**F2**	**F3**
Financial wellbeing (AB9)	**0.840**	Make financial choices (AD6)	**0.764**			Opportunities to meet goals (TR2)	**0.8504**		
Saving ability (AB8)	**0.624**	Overcome challenges (AD5)	**0.758**			Low-cost adult education (TR3)	**0.8498**		
Expense burden (AB7a-c)	**0.564**	Financial knowledge (AD12)	**0.697**			Easy to get around (TR6)	**0.7269**		
Income stability (AB1, AB2)	**0.430**	Budgeting skills (AD11)	**0.629**			Good job availability (TR1)	**0.7118**		
Housing stability (AB5, AB6)	**0.401**	Find ways to meet needs (AD4)	**0.619**			Low-cost healthcare (TR7)	**0.7001**		
Emergency cash options (AB11)	0.213	Job skills (AD10)	**0.513**			Transportation options (TR5)	**0.6846**		
Health insurance (AB10)	0.106	Job Barriers (AD14a-c)	**0.426**			Informed on issues (TR10)	**0.6628**		
Tangible social support (AB12)	−0.134	Internet use (AD3)	**0.422**			Community orgs. (TR9)	**0.6462**		
		Assistance barriers (AD13a-c)	**0.360**			Quality kids' schools (TR4)	**0.6211**		
		Stress inhibits planning (AD8)		**0.924**		Better living in future (TR19)		**0.9715**	
		Stress inhibits budgeting (AD7)		**0.838**		Afford needs in future (TR18)		**0.9363**	
		Stress inhibits goals (AD9)		**0.800**		Reach goals in future (TR20)		**0.8356**	
		Close social connections (AD1)			**0.957**	People help each other (TR15)			**0.8819**
		Confidants offer advice (AD2)			**0.734**	People get along (TR16)			**0.8521**
						People can be trusted (TR17)			**0.7714**
						Community is safe (TR8)			**0.4271**
						Discrimination (TR21)	0.1547	0.0418	0.2769

Bolded values indicate that the item was retained as part of the final measure.

Adaptive Capacity had a three-factor structure with items assessing financial efficacy, skills, and barriers (F1: Eigenvalue = 4.41; Cronbach's alpha = 0.84), financial stress (F2: Eigenvalue = 2.02; Cronbach's alpha = 0.90), and social support (F3: Eigenvalue = 0.97; Cronbach's alpha = 0.83). Item AD13a-c (0.360 factor loading) was retained even though it loaded <0.40 because it offers practical information about specific barriers households face in accessing “assistance and/or charity programs”. The full Adaptive Capacity measure (Mean score = 2.63; SD = 0.85; max range 0–5) with all included items had a Cronbach's alpha of 0.84.

Transformative Capacity had a three-factor structure with items assessing community services and resources (F1: Eigenvalue = 7.84; Cronbach's alpha = 0.92), household-level financial outlook (F2: Eigenvalue = 1.48; Cronbach's alpha = 0.95), and neighborhood cohesion and safety (F3: Eigenvalue = 1.18; Cronbach's alpha = 0.87). Item TR21 (which assesses perceived discrimination in various settings) did not load on any factors and was removed. The full Transformative Capacity measure (Mean score = 2.70; SD = 1.10; max range 0–5) with all included items had a Cronbach's alpha of 0.93.

Test bias was assessed by examining moderation effects between sample demographic characteristics and scores for the new measures. There was no test bias detected for the Absorptive Capacity or Adaptive Capacity measures by educational attainment, age, race/ethnicity, gender, or test mode. Education did moderate the relationship between the Transformative Capacity score and household food security status, but no other moderating effects were detected. Therefore, those using the Transformative Capacity measure with a sample from diverse educational backgrounds should assess the potential influence of educational attainment on the findings and consider controlling for this variable and interaction terms in analyses.

#### Convergent and discriminant validity

Spearman's correlation coefficients for the associations between the scores for the new measures and the validation variables indicated associations were largely in the expected directions (Table 3). All three new measures were positively associated with higher food security, better general health, and higher scores for the CD-RISC and CFPB FWS scales. All new measures were also negatively associated with experiencing a financial shock over the previous 12 months. Unexpectedly, there was statistically significant positive association between sports escapism and Adaptive Capacity.

**Table 3:** Spearman's correlation coefficients for assessing convergent and discriminant validity of the new measures.

	**Food security**	**General health**	**CD-RISC scores^a^**	**CFPB FWS scores^b^**	**Financial shock^c^**	**Sports escapism**
Absorptive capacity (*n* = 394)	0.550^*^	0.255^*^	0.231^*^	0.464^*^	−0.221^*^	0.004
Adaptive capacity (*n* = 325)	0.430^*^	0.320^*^	0.379^*^	0.474^*^	−0.253^*^	0.227^*^
Transformative capacity (*n* = 220)	0.310^*^	0.288^*^	0.384^*^	0.401^*^	−0.307^*^	0.193

* Statistically significant at the Bonferroni adjusted 0.0028 alpha level.

a Conner-Davidson Resilience Scale.

b Consumer Financial Protection Bureau's Financial Wellbeing Scale.

c Self-report of having experienced a household financial shock (e.g., job loss) in the previous 12 months.

#### Determining brief screener versions

For Absorptive Capacity, those who selected zero or one adults with income for AB1 and selected “Never” being able to save money for AB8 screened positive for “low” Absorptive Capacity with 89% sensitivity, 66% specificity, and Cohen's kappa of 0.503. While this screening combination was sensitive for detecting “low” Absorptive Capacity, the specificity and kappa scores were not ideal. This indicates that the Absorptive Capacity screener may have more false positives than desired (e.g., a household it categorized as “low” for Absorptive Capacity based on the screener but does not score “low” on the full measure). For Adaptive Capacity, those who selected “Strongly disagree,” “Disagree,” or “Slightly disagree” to either AD2 or AD4 screened positive for “low” Adaptive Capacity with 86% sensitivity, 73% specificity, and Cohen's kappa of 0.595. This measure was sensitive and only slightly under desired thresholds for specificity and kappa agreement scores. For Transformative Capacity, those who selected “Strongly disagree,” “Disagree,” or “Slightly disagree” to either TR6 or TR18 screened positive for “low” Transformative Capacity with 93% sensitivity, 82% specificity, and Cohen's kappa of 0.746. The Transformative Capacity screener scored well within desired thresholds for sensitivity, specificity, and kappa agreement scores. These screeners can be used for efficiently (i.e., by administering only two items instead of the full measures) assessing risk of “low” Absorptive, Adaptive, and Transformative Capacity, respectively, which may be useful for applications where administering the full measures is not feasible (e.g., intake screening to inform program/assistance referral).

## Discussion

Three new self-administered measures assessing household resilience were created. Each measure assesses a different aspect of household resilience to financial shocks, which are a major predictor for food insecurity. We envision these measures as independent tools to assess distinct aspects of household resilience, however, they can be used within the same survey for a comprehensive assessment of household resilience. The findings support reliability and validity of the new measures within a largely low-income and food insecure sample of households in the U.S. Higher scores for each of the measures were associated with greater food security, general health, general resilience, and financial wellbeing. Also, higher scores were negatively associated with having experienced a financial shock in the previous 12 months. Further, the new measures provide practical information that may be useful to those developing food insecurity interventions. For example, the Adaptive Capacity measure includes a list of barriers participants may face when looking for a job or accessing governmental assistance. This information can be crucial for tailoring intervention approaches to address frequently faced barriers among low-income populations. Also, while the sub-scales within each measure are not intended to be used independently, they can provide practical information as well. For example, if participants within a community score relatively lower on items within the Transformative Capacity measure that assesses community resources and services, this indicates that systems- and/or policy-based interventions may be needed to increase government spending and address infrastructure and resource shortfalls in that community. Finally, the brief screener versions of the capacity measures showed acceptable agreement with the full measures, which increases the potential broad applicability, especially in situations or settings where brief versions are the only feasible option (e.g., clinical intake screening and within Electronic Health Records).

Formative development work and psychometric testing in this study revealed that many, but not all, of the factors observed to be relevant to household resilience in the international literature were as relevant for a U.S. context. As a whole, the three measures contain items that assess key factors identified in the international literature, such as household income stability, expenditures, housing stability, social and human capital, psychosocial variables, perceived future outlook for the household, perceptions of transportation infrastructure, perceptions of safety, community social environment, and access to governmental assistance, health care, education/training, jobs, and services (23, 24, 27, 29). However, some factors observed in the international literature were not included in the final measures developed in this study. These included variables such as women's empowerment, perceptions of governance, access to informal safety nets (e.g., help from friends or family), quality of utilities infrastructure, environmental factors (e.g., soil quality, resource management, climate change, etc.), and technological uptake and advancement (23, 24, 27, 29). For these variables, either they did not emerge as relevant factors during the formative interviews (e.g., women's empowerment) and/or the items did not perform well in testing and were dropped (e.g., perceptions of governance). Further, this study examined at least two concepts not explicitly represented in the international literature—job barriers and discrimination. The job barriers items were included in the final measure of Absorptive Capacity and the topic of discrimination was covered within the job barriers items, but the community-level discrimination item was dropped due to factor analysis findings.

The Absorptive Capacity measure contains nine items assessing expense burden, income adequacy, and housing situation. These, and related variables, have been shown to be associated with food insecurity and negative impacts of household shocks (17, 45–47). Items that assessed medical insurance coverage and tangible social support/informal safety nets were dropped due to low factor loadings. Since unexpected medical costs are a common household financial shock (15) and medical insurance theoretically buffers these shocks (48), lacking this component may be a limitation of the measure. Similarly, items assessing tangible social support (such as help from friends or family with money, food, transportation, childcare, etc.) were dropped due to low factor loadings. Tangible social support has been shown to be positively associated with resilience, food security, and financial wellbeing (48–52). The item meant to assess tangible social support asked about the money, food, shelter, and other forms of support the household was currently receiving. The tangible social support item may not have loaded highly in the factor analysis because higher levels of currently receiving tangible social support may have been indicative of decreased financial wellbeing (i.e., The household is currently receiving money/food/shelter from friends or family because the household is not financially stable on its own). Instead, future measures should investigate items that assess the tangible social support the household has access to, should they need it, but is not currently utilizing.

The Adaptive Capacity measure contains eighteen items assessing financial efficacy, financial stress, social support, and adaptive barriers. These and related factors have been shown to be associated with food security, poverty, and ability to handle financial shocks (47, 48, 53–62). Unexpectedly, there was a statistically significant association between “sports escapism” and Adaptive Capacity. We hypothesized that there would be no association between “keeping up to date with sports…” and these capacity measures, but perhaps the social elements captured in the Absorptive Capacity (and Transformative Capacity) measures are also associated with households that are generally more socially connected, and following sports (e.g., viewing games with others) can be a social activity. However, investigating this association was beyond the scope of the current study.

The Transformative Capacity measure contains sixteen items assessing community resources, social environment, financial outlook, and access to opportunities. These and related factors have been shown to be associated with increased food security (3, 50, 51, 63–69). There were a high percentage of missing responses for the Transformative Capacity items. Many of the questions asked about aspects of the physical, social, and political/civic environment of the participant's community and many respondents selected “don't know”. Particularly, several of the questions related to perceived civic engagement of others had high missing percentages and were dropped. Areas in the U.S. affected by food insecurity are often relatively lacking in governmental services and resources but have limited political power and face barriers to civic engagement to change this situation (69–72). This may be a limitation of the measure and more research is needed to investigate the implications of lacking a robust assessment of civic engagement.

### Study limitations and strengths

The findings should be interpreted in the context of the study limitations. Firstly, this study presents a hypothesized scale based on an exploratory factor analysis. Additional work in future samples should be conducted to confirm the findings are generalizable beyond this sample. Further, this study utilized a convenience sample and may not be representative of food insecure households or households at risk for food insecurity in the U.S. The survey was offered in both online and paper formats, and respondents differed demographically by which mode they utilized. Allowing the paper survey, in addition to the online survey, may have reduced sampling error (e.g., allowing more of the target population to participate who did not have access to the internet), but may have increased measurement error (e.g., differences in interface between the modes and lack of automated skip logic for paper surveys) (73, 74). The rural U.S. was not well-represented in this study. While there was some rural representation in parts of WA and NC, the sample generally skewed urban with recruitment sites located in Tampa Bay (FL), San Diego (CA), Seattle (WA), and the Washington D.C. metro area. More research is needed to investigate potential differences in household resilience indicators in rural vs. urban contexts in the U.S. Also, men were not well-represented in the sample. Research has shown that men and women in the same household interpret and respond differently to the HFSSM questions (75, 76) and investigating potential gender differences for these new measures is needed. Another limitation is that we assessed household resilience cross-sectionally. The interactions between a shock and the resilience of a system are dynamic (23, 25, 28). Future longitudinal studies in the U.S. that follow households from before a shock and through the processes of exercising of their Absorptive, Adaptive, and Transformative Capacity, and the related implications for food security and health are warranted. Further, we assessed general financial shocks defined as either job loss or a large unexpected bill. Specific aspects of the financial shocks can modify the effect the shock has on the household, such as magnitude of the shock (25), types of shock (e.g., job loss vs. unexpected bill) (17), and if the household is experiencing only one or multiple simultaneous shocks (25). Future work to understand the relationship between the type of shock and impacts on Absorptive, Adaptive, and Transformative Capacity are warranted. Finally, the use of sports escapism to assess discriminant validity may not have been ideal. The variable was positively associated with adaptive and transformative capacity, seemingly due to the social connections that can come with following sports and that are assessed in those two measures. Also, it is possible people facing financial hardships might follow sports as a distraction, although, we would have expected to see associations in the opposite direction if that were true in this sample. We advise future studies to identify a different variable for assessing discriminant validity.

The strengths of this study are notable and include a robust formative phase incorporating evidence from the literature, experts, and individuals facing food insecurity, and comparison against validated and relevant measures to assess construct validity. Further, the sample was relatively large and diverse, such as by educational attainment, age, race/ethnicity, and represented five states across the U.S.

## Conclusion

This study describes the preliminary development of the first set of self-administered household resilience measures to assess Absorptive, Adaptive, and Transformative Capacities developed and evaluated among U.S. adults. Households with higher scores for the new measures may be more resilient to household-level financial shocks and less likely to suffer subsequent food insecurity. The measures are easily scored by simply taking a mean of included items, without the need for advanced coding or software. These tools, therefore, could be utilized by organizations that may have limited resources. Further, the measures provide not only a score, which can be useful for applications such as program evaluation and public health surveillance, but also include questions that provide practical information can be valuable to inform intervention development and needs assessments. Also, the brief screener versions can be useful in settings and applications where longer versions are not appropriate (e.g., clinical intake screening). Next steps for this work include disseminating these preliminary measures for others to confirm these findings in different samples, such as through confirmatory factor analysis and other approaches. Pending further testing, these tools have the potential to help those who design interventions to identify and target household resilience needs among populations to potentially reduce impact of financial shocks and ultimately help to mitigate food insecurity risk. Additionally, while conceptualized in the context of food insecurity, these measures may be broadly applicable to other related topics that could be explored in future studies. We envision these tools to be utilized by non-profit organizations, public health departments, hospitals and clinics, philanthropic organizations, social service organizations, researchers, and governmental organizations. Potential applications of the measures include needs assessments to inform intervention approaches, such as for Community Health Needs Assessments (CHNAs), program evaluation, clinical screening, research, and public health surveillance.

## Data availability statement

The raw data used in this study will be made available by the corresponding authors upon reasonable request.

## Ethics statement

The studies involving human participants were reviewed and approved by University of Nebraska Medical Center Institutional Review Board. The patients/participants provided their written informed consent to participate in this study.

## Author contributions

EC led the conception and implementation of the study, data analysis, manuscript drafting, and review process. LC led data collection efforts, assisted with manuscript drafting, review. TG assisted with data collection efforts, manuscript drafting, and review. JS assisted with developing the analysis approach, manuscript drafting, and review. AY provided scientific oversight, assisted with manuscript drafting, and review. All authors contributed to the article and approved the submitted version.
