# Supplementary material for: Development of three new multidimensional measures to assess household food insecurity resilience in the United States
Source: Front Public Health. 2022 Dec 14;10:1048501. doi: 10.3389/fpubh.2022.1048501 (PMC9794863; doi:10.3389/fpubh.2022.1048501)
Supplement: Supplementary file 1 [file Data_Sheet_1.docx]

**Supplementary Materials**

Development of three new multidimensional measures to assess household food insecurity resilience in the United States

Eric E. Calloway,^1^ PhD, RD, Leah R. Carpenter,^1^ MPH, Tony Gargano,^1^ MPH, Julia L. Sharp,^2^ PhD, & Amy L. Yaroch,^1^ PhD

1: The Gretchen Swanson Center for Nutrition, Omaha, NE, USA

2: Colorado State University, Fort Collins, CO, USA

**Email addresses of co-authors**:

Leah: lcarpenter@centerfornutrition.org

Tony: tgargano@centerfornutrition.org

Julia: jlsharp@colostate.edu

Amy: ayaroch@centerfornutrition.org

**Corresponding author**:

Eric E. Calloway

Research Scientist

Gretchen Swanson Center for Nutrition

14301 FNB Parkway, Suite 100

Omaha, NE 68154

Ph: 001-402-781-4943

Email: ecalloway@centerfornutrition.org

| **Supplementary Table 1. Scoring the Absorptive Capacity Measure** | | | | | |
| --- | --- | --- | --- | --- | --- |
| **Item ID** | **Response Options** | **Item Scoring** | | | **Measure Score** |
| AB1 | 0-10 | 0 Adults | 0 | Sum AB1 & AB2 score | **Mean of Scores** |
|  |  | 1 Adult | 1 |  |  |
|  |  | ≥2 Adults | 2 |  |  |
| AB2 | 0-10 (out of AB1 total) | 0 Adults | 0 |  |  |
|  |  | ≥1 Adult | 1 |  |  |
| AB5 | A) Housing that I/we own outright B) Housing that I/we pay to stay (e.g., rent, mortgage, or room fee) C) In friend’s or family’s housing, spare room, or couch (do not pay rent)  D) In a shelter, safe haven, or transitional housing E) In a car, van, or camper/RV on the street or parking lot F) Unsheltered on the street, a hallway or stairwell, or under a bridge, etc. | C-F | 0 | Sum AB5 & AB6 score |  |
|  |  | B | 1 |  |  |
|  |  | A | 2 |  |  |
| AB6 | No, Yes | Yes | 0 |  |  |
|  |  | No | 1 |  |  |
| AB7a | Housing payment (rent, mortgage, etc.) - Rental insurance or homeowner’s insurance - Utilities like electricity, gas, water, or other utilities - Internet, phone, and/or cable bills - Car payment, insurance, gas, car repair, or other transportation (Fees for the bus, subway, taxi/Uber) - Payments for credit card, student loans, or other loans and debt | Sum all responses from AB7a-AB7c that were selected | 9-18 | 0 |  |
| AB7b | Groceries, or store-bought food - Meals from restaurants - Pet expenses - Toiletries (such as shampoo, soap, toothpaste, other hygiene products) - Purchased clothing - Sending money to support family/friends |  | 6-8 | 1 |  |
| AB7c | Monthly insurance payments for health, dental, and/or vision care - Other medical costs like fees or co-pays, medicine, medical equipment, etc. - Cost of child’s activities (e.g., sports, dance, tutoring, etc.) - Childcare, or children’s school fees - Costs for care for an adult or child with disabilities or an elderly adult - Child support or spousal support payments |  | 3-5 | 2 |  |
|  |  |  | 0-2 | 3 |  |
| AB8 | A) Never B) Only 1 or 2 months C) Some months but not every month D) Every month or almost every month | A |  | 0 |  |
|  |  | B |  | 1 |  |
|  |  | C |  | 2 |  |
|  |  | D |  | 3 |  |
| AB9 | A) Not getting by B) Just barely getting by C) Doing okay D) Living comfortably | A |  | 0 |  |
|  |  | B |  | 1 |  |
|  |  | C |  | 2 |  |
|  |  | D |  | 3 |  |

| **Supplementary Table 2. Scoring the Adaptive Capacity Measure** | | | | | |
| --- | --- | --- | --- | --- | --- |
| **Item ID** | **Response Options** | **Item Scoring** | | | **Measure Score** |
| AD1 |  |  |  |  | **Mean of Item Scores** |
| AD2 | Strongly disagree |  |  | 0 |  |
| AD3 | Disagree |  |  | 1 |  |
| AD4 | Slightly disagree |  |  | 2 |  |
| AD5 | Slightly agree |  |  | 3 |  |
| AD6 | Agree |  |  | 4 |  |
| AD10 | Strongly agree |  |  | 5 |  |
| AD11 |  |  |  |  |  |
| AD12 |  |  |  |  |  |
|  | Strongly disagree |  |  | 5 |  |
| AD7 | Disagree |  |  | 4 |  |
| AD8 | Slightly disagree |  |  | 3 |  |
| AD9 | Slightly agree |  |  | 2 |  |
|  | Agree |  |  | 1 |  |
|  | Strongly agree |  |  | 0 |  |
| AD13a | Make too much to qualify - Would qualify for only a small amount, not worth it - Currently using all that my household is eligible for - Disqualified due to things in my/our past - Do not want it to impact immigration status | Sum all responses from AB13a-AB13c that were selected | 5-14 | 0 |  |
|  |  |  | 4 | 1 |  |
| AD13b | Do not know much about assistance programs or charities available - Takes too much time and effort to apply or sign-up - Too confusing to apply or sign-up - Hard to travel to apply, sign-up, or use services - Hard to use the internet or phone to apply, sign-up, or use services |  | 3 | 2 |  |
|  |  |  | 2 | 3 |  |
| AD13c | Do not like relying on assistance programs or charities - Do not want them to have my/our information - Want to save it for others that need it more - Do not like how assistance programs or charities treat people seeking help |  | 1 | 4 |  |
|  |  |  | 0 | 5 |  |
| Ad14a | Physical or mental health condition, disability, or injury - Discrimination - Experience domestic violence - Fear of losing or reducing government benefits - Need to care for an adult or child with disabilities, or an elderly adult - Concerns about contact with police or government agents | Sum all responses from AB13a-AB13c that were selected | 5-20 | 0 |  |
|  |  |  | 4 | 1 |  |
| Ad14b | Lack of reliable transportation - Lack affordable of childcare or day care - Limited time available - Lack of work clothing - Difficulty maintaining hygiene - Not having a permanent address, email, or phone number - Addiction to drugs or alcohol - Past criminal record |  | 3 | 2 |  |
|  |  |  | 2 | 3 |  |
| Ad14c | Cannot speak English well - Cannot read or write English well - Lack of experience creating a résumé or interviewing - Lack of computer skills - Lack of needed education - Lack of job skills or experience |  | 1 | 4 |  |
|  |  |  | 0 | 5 |  |
|  | | | | | |

| **Supplementary Table 3. Scoring the Transformative Capacity Measure** | | | |
| --- | --- | --- | --- |
| **Item ID** | **Response Options** | **Item Scoring** | **Measure Score** |
| TR1-TR10, TR15-TR20 | Strongly Disagree | 0 | Mean of Item Scores |
|  | Disagree | 1 |  |
|  | Slightly disagree | 2 |  |
|  | Slightly agree | 3 |  |
|  | Agree | 4 |  |
|  | Strongly agree | 5 |  |

| **Supplementary Table 4. Items and response options for the new household resilience measures – Absorptive capacity, Adaptive capacity, and Transformative capacity** | | | | | |
| --- | --- | --- | --- | --- | --- |
| **Measure** | **Sub-scale/Sub-topic** | **Item Number From Testing** | **Item Name** | **Item** | **Response Options** |
| Absorptive Capacity | Absorptive Capacity | AB1 | Adults received income | How many adults in your household received income last month that was used for household expenses?  (Count any income such as from a job, assistance program, retirement program, or any other income that came to the household) | 0-10, Don't know |
|  |  | AB2 | Adults lost income | In the past 12 months, how many adults in your household lost their job, lost a source of income, or had their hours cut?   (Count any income such as from a job, assistance program, retirement program, or any other income that came to the household) | 0-10 (out of total receiving), Don't know |
|  |  | AB5 | Housing situation | Where does your household live? | Housing that I/we own outright - Housing that I/we pay to stay (e.g., rent, mortgage, or room fee) - In friend’s or family’s housing, spare room, or couch (do not pay rent) - In a shelter, safe haven, or transitional housing - In a car, van, or camper/RV on the street or parking lot - Unsheltered on the street, a hallway or stairwell, or under a bridge, etc. |
|  |  | AB6 | Forced to leave | In the past 12 months, has your household had to move due to foreclosure, eviction, or being forced to leave? | No - Yes - Don't know |
|  |  | AB7a | Expenses part A | Part A: Last month, which bills or expenses were hard to afford?  (Select all that apply) | Housing payment (rent, mortgage, etc.) - Rental insurance or homeowner’s insurance - Utilities like electricity, gas, water or other utilities - Internet, phone, and/or cable bills - Car payment, insurance, gas, car repair, or other transportation (Fees for the bus, subway, taxi/Uber) - Payments for credit card, student loans, or other loans and debt - None of the above |
|  |  | AB7b | Expenses part B | Part B: Last month, which bills or expenses were hard to afford?  (Select all that apply) | Groceries, or store-bought food - Meals from restaurants - Pet expenses - Toiletries (such as shampoo, soap, toothpaste, other hygiene products) - Purchased clothing - Sending money to support family/friends - None of the above |
|  |  | AB7c | Expenses part C | Part C: Last month, which bills or expenses were hard to afford?  (Select all that apply) | Monthly insurance payments for health, dental, and/or vision care - Other medical costs like fees or co-pays, medicine, medical equipment, etc. - Cost of child’s activities (e.g., sports, dance, tutoring, etc.) - Childcare, or children’s school fees - Costs for care for an adult or child with disabilities or an elderly adult - Child support or spousal support payments - None of the above |
|  |  | AB8 | Saving ability | How often in the past 12 months was your household able to put money into savings? | Never - Only 1 or 2 months - Some months but not every month - Every month or almost every month - Don't know |
|  |  | AB9 | Financial wellbeing | Which best describes how well your household is doing with finances? | Not getting by - Just barely getting by - Doing okay - Living comfortably - Don't know |
| Adaptive Capacity | Financial efficacy, skills, and barriers | AD3 | Internet use | If needed, it is easy for (me/us) to use the internet to search for assistance programs, find jobs, and/or complete online application forms. | Strongly disagree - Disagree - Slightly disagree - Slightly agree - Agree - Strongly agree - Don't know |
|  |  | AD4 | Find ways to meet need | If (I/we) have a financial challenge, I believe (I/we) can find ways to get what (I/we) need. | Strongly disagree - Disagree - Slightly disagree - Slightly agree - Agree - Strongly agree - Don't know |
|  |  | AD5 | Overcome challenges | I believe (I/we) can overcome most financial challenges. | Strongly disagree - Disagree - Slightly disagree - Slightly agree - Agree - Strongly agree - Don't know |
|  |  | AD6 | Make financial choices | (I/We) can figure out what choices to make when (I/we) face important financial challenges. | Strongly disagree - Disagree - Slightly disagree - Slightly agree - Agree - Strongly agree - Don't know |
|  |  | AD10 | Job skills | (I/We) have work skills and job experience that would be helpful if (I/we) needed a job or another job. | Strongly disagree - Disagree - Slightly disagree - Slightly agree - Agree - Strongly agree - Don't know |
|  |  | AD11 | Budgeting skills | (I/We) can use a spending plan or budget to adjust spending if needed. | Strongly disagree - Disagree - Slightly disagree - Slightly agree - Agree - Strongly agree - Don't know |
|  |  | AD12 | Financial knowledge | I feel (I/we) are well informed about financial matters. | Strongly disagree - Disagree - Slightly disagree - Slightly agree - Agree - Strongly agree - Don't know |
|  |  | AD13a | Assistance barriers part A | Part A. Would any of the following keep your household from using assistance programs or charities, or for applying for more assistance (e.g., food stamps, food banks or other charities, Medicaid, etc.)? (Select all that apply) | Make too much to qualify - Would qualify for only a small amount, not worth it - Currently using all that my household is eligible for - Disqualified due to things in my/our past - Do not want it to impact immigration status - None of the above |
|  |  | AD13b | Assistance barriers part B | Part B. Would any of the following keep your household from using assistance programs or charities, or for applying for more assistance (e.g., food stamps, food banks or other charities, Medicaid, etc.)? (Select all that apply) | Do not know much about assistance programs or charities available - Takes too much time and effort to apply or sign-up - Too confusing to apply or sign-up - Hard to travel to apply, sign-up, or use services - Hard to use the internet or phone to apply, sign-up, or use services - None of the above |
|  |  | AD13c | Assistance barriers part C | Part C. Would any of the following keep your household from using assistance programs or charities, or for applying for more assistance (e.g., food stamps, food banks or other charities, Medicaid, etc.)? (Select all that apply) | Do not like relying on assistance programs or charities - Do not want them to have my/our information - Want to save it for others that need it more - Do not like how assistance programs or charities treat people seeking help - None of the above |
|  |  | Ad14a | Job barriers part A | Part A. Which of the following would make it hard for you or any adult in your household to get a job, or add another job, if needed?  (Select all that apply) | Physical or mental health condition, disability, or injury - Discrimination - Experience domestic violence - Fear of losing or reducing government benefits - Need to care for an adult or child with disabilities, or an elderly adult - Concerns about contact with police or government agents - None of the above |
|  |  | Ad14b | Job barriers part B | Part B. Which of the following would make it hard for you or any adult in your household to get a job, or add another job, if needed?  (Select all that apply) | Lack of reliable transportation - Lack affordable of childcare or day care - Limited time available - Lack of work clothing - Difficulty maintaining hygiene - Not having a permanent address, email, or phone number - Addiction to drugs or alcohol - Past criminal record - None of the above |
|  |  | Ad14c | Job barriers part C | Part C. Which of the following would make it hard for you or any adult in your household to get a job, or add another job, if needed?  (Select all that apply) | Cannot speak English well - Cannot read or write English well - Lack of experience creating a résumé or interviewing - Lack of computer skills - Lack of needed education - Lack of job skills or experience - None of the above |
|  | Financial Stress | AD7 | Stress inhibits budgeting | The financial stress (I/we) feel makes it hard to focus on budgeting. | Strongly disagree - Disagree - Slightly disagree - Slightly agree - Agree - Strongly agree - Don't know |
|  |  | AD8 | Stress inhibits planning | The financial stress (I/we) feel makes it hard to focus on planning. | Strongly disagree - Disagree - Slightly disagree - Slightly agree - Agree - Strongly agree - Don't know |
|  |  | AD9 | Stress inhibits goals | The financial stress (I/we) feel makes it hard to focus on working towards life goals. | Strongly disagree - Disagree - Slightly disagree - Slightly agree - Agree - Strongly agree - Don't know |
|  | Social support | AD1 | Close social connections | (I/We) can get helpful advice from others when dealing with a financial problem. | Strongly disagree - Disagree - Slightly disagree - Slightly agree - Agree - Strongly agree - Don't know |
|  |  | AD2 | Confidants offer advice | (I/We) know people personally who (I/we) can discuss things with like assistance programs, charitable organizations, and/or jobs or educational opportunities. | Strongly disagree - Disagree - Slightly disagree - Slightly agree - Agree - Strongly agree - Don't know |
| Transformative Capacity | Community services and resources | TR1 | Good job availability | There are options available for adults in my community to earn a good living. | Strongly disagree - Disagree - Slightly disagree - Slightly agree - Agree - Strongly agree - Don't know |
|  |  | TR2 | Opportunities to meet goals | Households in my community have opportunities to reach financial goals and/or save money for the future. | Strongly disagree - Disagree - Slightly disagree - Slightly agree - Agree - Strongly agree - Don't know |
|  |  | TR3 | Low-cost adult education | There are good affordable educational, job training, and/or professional licensing options for adults in my community (e.g., trade school, community college, university, or other similar education). | Strongly disagree - Disagree - Slightly disagree - Slightly agree - Agree - Strongly agree - Don't know |
|  |  | TR4 | Quality kids' schools | There are good affordable schools available to children of all ages in my community (e.g., preschools, childcare centers, public schools, charter schools, etc.). | Strongly disagree - Disagree - Slightly disagree - Slightly agree - Agree - Strongly agree - Don't know |
|  |  | TR5 | Transportation options | There are good public transportation options in my community. | Strongly disagree - Disagree - Slightly disagree - Slightly agree - Agree - Strongly agree - Don't know |
|  |  | TR6 | Easy to get around | It is easy for people in my community to travel around to the places they need to be. | Strongly disagree - Disagree - Slightly disagree - Slightly agree - Agree - Strongly agree - Don't know |
|  |  | TR7 | Low-cost healthcare | There are good affordable healthcare services in my community. | Strongly disagree - Disagree - Slightly disagree - Slightly agree - Agree - Strongly agree - Don't know |
|  |  | TR9 | Community organizations | There are plenty of organizations in my community that help households in need. | Strongly disagree - Disagree - Slightly disagree - Slightly agree - Agree - Strongly agree - Don't know |
|  |  | TR10 | Informed on issues | People in my community stay informed about community issues. | Strongly disagree - Disagree - Slightly disagree - Slightly agree - Agree - Strongly agree - Don't know |
|  | Household-level financial outlook | TR18 | Afford needs in future | In the next five years, I believe my household will be better able to afford basic needs such as food, housing, utilities, transportation, and medical care. | Strongly disagree - Disagree - Slightly disagree - Slightly agree - Agree - Strongly agree - Don't know |
|  |  | TR19 | Better living in future | In the next five years, I believe my household will be better able to earn a good living. | Strongly disagree - Disagree - Slightly disagree - Slightly agree - Agree - Strongly agree - Don't know |
|  |  | TR20 | Reach goals in future | In the next five years, I believe my household will be better able to reach financial goals and/or save money for the future. | Strongly disagree - Disagree - Slightly disagree - Slightly agree - Agree - Strongly agree - Don't know |
|  | Neighborhood cohesion and safety | TR8 | Community is safe | My community is safe. | Strongly disagree - Disagree - Slightly disagree - Slightly agree - Agree - Strongly agree - Don't know |
|  |  | TR15 | People help each other | People in my community are willing to help their neighbors. | Strongly disagree - Disagree - Slightly disagree - Slightly agree - Agree - Strongly agree - Don't know |
|  |  | TR16 | People get along | People in my community usually get along with each other. | Strongly disagree - Disagree - Slightly disagree - Slightly agree - Agree - Strongly agree - Don't know |
|  |  | TR17 | People can be trusted | People in my community can be trusted. | Strongly disagree - Disagree - Slightly disagree - Slightly agree - Agree - Strongly agree - Don't know |
